# Correlation of the gamma passing rates with the differences in the dose-volumetric parameters between the original VMAT plans and actual deliveries of the VMAT plans

**DOI:** 10.1371/journal.pone.0244690

**Published:** 2020-12-29

**Authors:** Jong Min Park, Chang Heon Choi, Hong-Gyun Wu, Jung-in Kim

**Affiliations:** 1 Department of Radiation Oncology, Seoul National University Hospital, Seoul, Korea; 2 Institute of Radiation Medicine, Seoul National University Medical Research Center, Seoul, Korea; 3 Biomedical Research Institute, Seoul National University Hospital, Seoul, Korea; 4 Department of Radiation Oncology, Seoul National University College of Medicine, Seoul, Korea; University of Nebraska Medical Center, UNITED STATES

## Abstract

**Purpose:**

The aim of this study was to investigate the correlations of the gamma passing rates (GPR) with the dose-volumetric parameter changes between the original volumetric modulated arc therapy (VMAT) plans and the actual deliveries of the VMAT plans (DV errors). We compared the correlations of the TrueBeam STx system to those of a C-series linac.

**Methods:**

A total of 20 patients with head and neck (H&N) cancer were retrospectively selected for this study. For each patient, two VMAT plans with the TrueBeam STx and Trilogy (C-series linac) systems were generated under similar modulation degrees. Both the global and local GPRs with various gamma criteria (3%/3 mm, 2%/2 mm, 2%/1 mm, 1%/2 mm, and 1%/1 mm) were acquired with the 2D dose distributions measured using the MapCHECK2 detector array. During VMAT deliveries, the linac log files of the multi-leaf collimator positions, gantry angles, and delivered monitor units were acquired. The DV errors were calculated with the 3D dose distributions reconstructed using the log files. Subsequently, Spearman’s rank correlation coefficients (*r*_*s*_) and the corresponding *p* values were calculated between the GPRs and the DV errors.

**Results:**

For the Trilogy system, the *r*_*s*_ values with *p* < 0.05 showed weak correlations between the GPRs and the DV errors (*r*_*s*_<0.4) whereas for the TrueBeam STx system, moderate or strong correlations were observed (*r*_*s*_≥0.4). The DV errors in the V_20Gy_ of the left parotid gland and those in the mean dose of the right parotid gland showed strong correlations (always with *r*_*s*_ > 0.6) with the GPRs with gamma criteria except 3%/3 mm. As the GPRs increased, the DV errors decreased.

**Conclusion:**

The GPRs showed strong correlations with some of the DV errors for the VMAT plans for H&N cancer with the TrueBeam STx system.

## Introduction

The most popular method of pre-treatment patient-specific quality assurance (QA) for intensity modulated radiation therapy (IMRT) or volumetric modulated arc therapy (VMAT) in the clinic is the gamma index method proposed by Low *et al*. [1]. The gamma index method can effectively identify and quantify differences in the two dose distributions [2,3]. However, several studies have questioned the clinical relevance of gamma passing rates (GPRs) [4,5]. Nelms *et al*. demonstrated that there is a lack of correlation between the GPRs and clinically relevant dose-volumetric parameter changes between plans and deliveries (DV errors) by utilising a total of 24 IMRT plans generated with a C-series linac [4]. Similarly, Stasi *et al*. showed that there were weak correlations between the GPRs and the DV errors of clinically relevant DV endpoints by utilising 27 prostate and 15 head and neck (H&N) IMRT plans [5]. They also showed cases where high GPRs did not necessarily indicate good consistency in anatomy dose metrics (*i*.*e*., false negatives) [5]. In this respect, several studies suggested log-file-based pre-treatment QA or calculation of the modulation indices as a pre-treatment patient-specific QA method for IMRT or VMAT [6–10]. However, these methods have a limitation in that they are not based on independent dose measurements; therefore, the gamma evaluation is still widely adopted in the clinic as a verification method of IMRT and VMAT plans.

The previous studies demonstrated the clinical irrelevance of the GPRs of IMRT plans with a C-series linac [4,5]; however, no study has been performed with the TrueBeam STx system (Varian Medical Systems, Palo Alto, CA, USA), which delivers treatment plans more accurately than the C-series linac by using an integrated control system which is called supervisor. It also has a greater advanced log-file generation capability than that of the C-series linac. Park *et al*. demonstrated that the GPRs of VMAT plans with the C-series linac were different from those with the TrueBeam STx system although both the VMAT plans were generated under identical conditions [11]. In other words, although both the VMAT plans were generated with an identical treatment planning system using identical patient computed tomography (CT) images, structure sets, prescription doses, and normal tissue tolerance levels, and GPRs were acquired using the same dosimeter, the GPRs with the TrueBeam STx could be different from those with the C-series linac [11]. This might be attributed to the difference in the modulation degree between the TrueBeam STx and C-series linac plans, or the difference in the operation mechanisms of the TrueBeam STx system and the C-series linac. In addition, it is unclear whether the predictive power of the GPRs with the TrueBeam STx regarding the accuracy of VMAT delivery is also as poor as that with the C-series linac. Therefore, in the present study, we investigated the correlations of the GPRs with the DV errors of clinically relevant DV end points with the TrueBeam STx compared with those with the C-series linac utilising a total of 20 VMAT plans.

## Materials and methods

### Patient selection and simulation

After receiving approval from the institutional review board (IRB), a total of 20 patients with nasopharyngeal cancer (H&N cancer) treated using the VMAT technique were retrospectively selected for this study. Approval for this study was obtained from the institutional review board of Seoul National University Hospital (IRB No. 1901-059-1002). This study is a retrospective study using an anonymized patient’s CT image set and treatment plan, which cause minimal risk to the patient. Therefore, this study was granted exemption for informed consent from IRB. Each patient underwent CT scans using the Brilliance CT Big Bore^TM^ system with a slice thickness of 3 mm (Phillips, Amsterdam, Netherlands) in the supine position. Each patient was immobilised using a thermoplastic mask and a Silverman pillow (Bionix Radiation Therapy, Toledo, OH).

### Volumetric modulated arc therapy plans

For each patient, two VMAT plans were generated: one using the Trilogy^TM^ system with the Millennium 120^TM^ multi-leaf collimator (MLC), which is a C-series linac, and the other using the TrueBeam STx^TM^ system with the high-definition (HD) 120^TM^ MLC (Varian Medical Systems, Palo Alto, CA). For both plans of each patient, the CT image set, structure set, prescription doses, and set of dose-volume constraints used for planning were identical. The simultaneous integrated boost technique was used with three planning target volumes (PTVs), which were PTV_67.5_ (prescription dose = 67.5 Gy and daily dose = 2.25 Gy), PTV_54_ (prescription dose = 54 Gy and daily dose = 1.8 Gy), and PTV_48_ (prescription dose = 48 Gy and daily dose = 1.6 Gy). These prescription doses were delivered in 30 fractions. Each plan in the present study was generated with the Eclipse^TM^ system (Varian Medical Systems, Palo Alto, CA) using 6 MV photon beams and two full arcs. For optimisation, the progressive resolution optimizer (PRO3, ver.13, Varian Medical Systems, Palo Alto, CA) was used. During optimisation, identical dose-volume constraints based on the Quantitative Analyses of Normal Tissue Effects in the Clinic (QUANTEC) recommendations were used for both the VMAT plans with the Trilogy and TrueBeam STx systems [12]. After optimisation, dose distributions were calculated (dose calculation resolution = 1 mm) using the anisotropic analytic algorithm (AAA, ver. 13, Varian Medical Systems, Palo Alto, CA). After dose calculation, each VMAT plan in the present study was normalised to cover 95% of the PTV_67.5_ with 95% of the prescription dose of 67.5 Gy. A total of 46 clinically relevant dose-volumetric parameters were calculated for each VMAT plan. For each PTV, the dose received by at least 99% of the structure volume (D_99%_), D_98%_, D_95%_, D_50%_, D_5%_, D_2%_, D_1%_, minimum dose, maximum dose, and mean dose were calculated. For both the left and right parotid glands (PGs), the volumes irradiated by at least 20 Gy (V_20Gy_), V_50%_, and mean doses were calculated. For the optic chiasm, both the left and right optic nerves, and both the left and right lenses, the maximum doses were calculated. For the spinal cord and brain stem, the maximum doses were calculated. For body, the values of V_100%_ and V_50%_, and mean doses were calculated. To investigate the modulation degrees of the VMAT plans with the Trilogy and TrueBeam STx systems, the modulation complexity score for VMAT (MCS_v_) and the leaf travel modulation complexity score (LTMCS) were calculated for each VMAT plan [13].

### Gamma index method

For the gamma evaluation, a MapCHECK2^TM^ detector array inserted in a MapPHAN^TM^ (Sun Nuclear Corporation, Melbourne, FL) was utilised for the measurements of 2D planar dose distributions of VMAT plans. To determine the reference dose distributions of each VMAT plan, a CT image set of MapCHECK2 in the MapPHAN was acquired with a slice thickness of 1 mm and the CT number of that structure (the MapPHAN including MapCHECK2) was assigned as 455 according to the manufacturer’s guideline [2]. Utilising this CT image set, verification plans of each VMAT plan were generated in the Eclipse system. The reference 2D dose distributions were calculated with a dose calculation grid size of 1 mm, which is the finest dose calculation grid size of the Eclipse system. Before the measurements with the MapCHECK2 detector array, the outputs of the Trilogy and TrueBeam STx systems were calibrated according to the American Association of Physicists in Medicine (AAPM) Task Group 51 (TG-51) protocol [14]. The MapCHECK2 detector array was also calibrated according to the manufacturer’s guideline before the measurements of 2D dose distributions of VMAT plans [2]. The setup of the MapCHECK2 dosimeter was verified by acquiring the cone beam computed tomography (CBCT) images of the Trilogy and TrueBeam STx systems. Using the SNC patient^TM^ software (Sun Nuclear Corporation, Melbourne, FL), both the global and local gamma evaluations with absolute doses were performed with various gamma criteria of 3%/3 mm, 2%/2 mm, 2%/1 mm, 1%/2 mm, and 1%/1 mm. When performing gamma evaluation, the threshold value was 10%.

### Differences in the dose-volumetric parameters between the original VMAT plans and the VMAT plans reconstructed with the log files

During measurements with the MapCHECK2 detector array, the log files recorded in the linac control system during VMAT deliveries were acquired with both the Trilogy and TrueBeam STx systems. The log files were records of the actual MLC positions, gantry angles, and delivered monitor units (MUs) during VMAT delivery [15]. Using an in-house program written in MATLAB (ver.8.1, MathWorks Inc., Natick, MA), the log files were combined and formatted to correspond to the VMAT plan file in DICOM-RT format and this plan file was imported to the Eclipse system. Subsequently, 3D dose distribution was calculated with a CT image set identical to that used to generate the original VMAT plan. The dose calculation resolution was identical to that of the original VMAT plan, which was 1 mm. With a structure set identical to that of the original VMAT plan, a total of 46 clinically relevant dose-volumetric parameters were calculated, which were the same as those calculated with the original VMAT plans. Subsequently, the DV errors were calculated.

### Differences in the GPRs between the original VMAT plans and the VMAT plans reconstructed with the log files (GPR_cal_)

To evaluate the changes in the doses between the original VMAT plans and the VMAT plans reconstructed with the log files, we performed gamma evaluations between the original VMAT plans and the VMAT plans reconstructed with the log files. Since there were an enormous number of points of doses to be evaluated with the gamma-index method in the case of the 3D gamma evaluation on the patient’s whole body, which potentially results in underestimation of the changes in the GPR values, we performed 2D gamma evaluation as described above instead of 3D gamma evaluation. In other words, for both the original VMAT plans and the VMAT plans reconstructed with the log files, 2D dose distributions were calculated utilising the CT image set of the MapPHAN with the MapCHECK2 and the values of the GPR_cal_ (GPRs with the 2D dose distribution calculated with the original VMAT and that calculated with the VMAT reconstructed with the log files) were acquired.

### Correlations between the GPRs and the DV errors

The correlations between the GPRs with various gamma criteria (calculated vs. measured) and the DV errors were analysed by calculating Spearman’s rank correlation coefficients (*r*_*s*_) with the corresponding *p* values. The *r*_*s*_ values with *p* values equal to or less than 0.05 were regarded as statistically significant in the present study. Following the Evans guidelines proposed in 1996, the absolute *r*_*s*_ values equal to or larger than 0.2 and smaller than 0.4 indicate weak correlations (0.2 ≤ *r*_*s*_ < 0.4); The absolute *r*_*s*_ values equal to or larger than 0.4 and smaller than 0.6 indicate moderate correlations (0.4 ≤ *r*_*s*_ < 0.6); The absolute *r*_*s*_ values equal to or larger than 0.6 and smaller than 0.8 indicate strong correlations (0.6 ≤ *r*_*s*_ < 0.8); The absolute *r*_*s*_ values equal to or larger than 0.8 indicate very strong correlations (r ≥ 0.8) [16].

## Results

### GPRs of the Trilogy and TrueBeam STx systems

The global and local GPRs with the gamma criteria of 3%/3 mm, 2%/2 mm, 2%/1 mm, 1%/2 mm, and 1%/1 mm as well as the values of MCS_v_ and LTMCS are shown in Table 1. According to the previous studies and guidelines, each VMAT plan in the present study was clinically acceptable based on the QA threshold as the global GPRs with 2%/2 mm were always higher than 90% [17,18]. Except the GPRs with 3%/3 mm, both the global and local GPRs of the VMAT plans with the TrueBeam STx system were always higher than those with the Trilogy system with statistical significance (*p* ≤ 0.05). Consistently, the average MLC positioning error, gantry angle error, and the MU delivery error during VMAT delivery with the TrueBeam STx system were 0.09 ± 0.01 mm, 0.03° ± 0.00°, and 0.02 ± 0.01 MU, respectively, whereas those with the Trilogy system were 0.19 ± 0.06 mm, 0.05° ± 0.00°, and 0.11 ± 0.09 MU, respectively. However, the values of the MCS_v_ and LTMCS indicated no statistically significant differences in the modulation degrees between the VMAT plans with the TrueBeam STx and Trilogy systems (*p* > 0.05). The values of MCS_v_ and LTMCS indicated that the modulation degrees of both VMAT plans with the TrueBeam STx and Trilogy systems were high as the values of MCS_v_ and LTMCS vary from 0 to 1 and these values decrease with the increase in the modulation degree [13].

**Table 1:** Global and local gamma passing rates with various gamma criteria and modulation degrees of the head and neck VMAT plans with the C-series linac and the TrueBeam STx.

Gamma criterion	C-series linac	TrueBeam STx	*p*
Global gamma passing rates (%)			
3%/3 mm	99.44 ± 0.60	99.67 ± 0.36	0.063
2%/2 mm	96.27 ± 2.40	97.46 ± 1.57	*0*.*026*
2%/1 mm	90.15 ± 3.88	92.18 ± 3.69	*0*.*031*
1%/2 mm	87.76 ± 5.21	89.88 ± 3.07	*0*.*050*
1%/1 mm	72.72 ± 6.86	75.63 ± 5.56	*0*.*049*
Local gamma passing rates (%)			
3%/3 mm	93.55 ± 1.87	93.59 ± 1.54	0.464
2%/2 mm	85.11 ± 4.13	86.85 ± 2.62	*0*.*048*
2%/1 mm	67.27 ± 5.59	70.02 ± 4.23	*0*.*031*
1%/2 mm	80.08 ± 5.58	82.77 ± 3.22	*0*.*027*
1%/1 mm	57.96 ± 6.31	61.40 ± 4.60	*0*.*019*
Modulation degree			
MCS_v_	0.0012 ± 0.0003	0.0013 ± 0.0003	0.092
LTMCS	0.0004 ± 0.0002	0.0005 ± 0.0002	0.079

VMAT: Volumetric modulated arc therapy, MCS_v_: Modulation complexity score for VMAT, LTMCS: Leaf travel modulation complexity score.

### GPR_cal_ between the original VMAT plans and the VMAT plans reconstructed with the log files

The values of the global and local GPR_cal_ with the gamma criteria of 3%/3 mm, 2%/2 mm, 2%/1 mm, 1%/2 mm, and 1%/1 mm between the original VMAT plans and the VMAT plans reconstructed with the log files are shown in Table 2. The statistically significant differences in the GPR_cal_ values between the original VMAT plans and the VMAT plans reconstructed with the log files were observed at the global gamma evaluation with 2%/1 mm and local gamma evaluations with 1%/2 mm and 1%/1 mm. For both global and local GPR_cal_, the differences between the GPR_cal_ with 3%/3 mm and those with 1%/1 mm of the TrueBeam STx system were higher than those of the Trilogy system.

**Table 2:** Global and local gamma passing rates between the original VMAT plans and the VMAT plans reconstructed with the log files (GPR_cal_) with various gamma criteria.

Gamma criterion	C-series linac	TrueBeam STx	*p*
Global GPR_cal_ (%)			
3%/3 mm	99.91 ± 0.32	100.00 ± 0.02	0.121
2%/2 mm	99.86 ± 0.37	99.99 ± 0.05	0.079
2%/1 mm	99.73 ± 0.48	99.92 ± 0.11	*0*.*042*
1%/2 mm	99.72 ± 0.54	99.64 ± 0.23	0.268
1%/1 mm	99.09 ± 1.54	98.67 ± 0.73	0.129
Local GPR_cal_ (%)			
3%/3 mm	99.82 ± 0.42	99.91 ± 0.22	0.189
2%/2 mm	99.58 ± 0.81	99.52 ± 0.65	0.385
2%/1 mm	98.20 ± 3.04	98.04 ± 2.18	0.416
1%/2 mm	99.15 ± 1.60	98.12 ± 1.25	*0*.*008*
1%/1 mm	96.06 ± 5.47	93.42 ± 3.45	*0*.*029*

VMAT: Volumetric modulated arc therapy.

### Correlations between the GPRs and the DV errors of the Trilogy system

The average values of each DV error with the Trilogy system are shown in S1 Table. Only the statistically significant correlation coefficients between the GPRs and the DV errors with the Trilogy system are shown in Table 3 with the corresponding *p* values. Every correlation coefficients between the GPRs and the DV errors are shown in S2 Table. Among a total of 46 dose-volumetric parameters evaluated in this study, both the global and local GPRs with 2%/1 mm showed statistically significant correlations with the DV errors most frequently although both the global and local GPRs showed only six *r*_*s*_ values with *p* values less than 0.05 from a total of 46 *r*_*s*_ values. The highest correlation was observed between the local GPR with 1%/1 mm and the DV error in the D_95%_ of PTV_54_ (*r*_*s*_ = -0.463 with *p* = 0.003). In most cases with the Trilogy system (*r*_*s*_ with *p* < 0.05), weak correlations were observed between the GPRs and the DV errors (absolute values of *r*_*s*_ < 0.4). Moderate correlations were rarely observed between the GPRs and DV errors (four cases showing 0.4 < absolute values of *r*_*s*_ < 0.5).

**Table 3:** Correlations of the gamma passing rates with the changes in the dose-volumetric parameters between the original VMAT plans and the plans reconstructed with the log files acquired during the VMAT deliveries of the C-series linac.

DV parameter	3%/3 mm		2%/2 mm		2%/1 mm		1%/2 mm		1%/1 mm	
	*r*_*s*_	*p*	*r*_*s*_	*P*	*r*_*s*_	*p*	*r*_*s*_	*p*	*r*_*s*_	*P*
Global gamma passing rates										
PTV_48_ D_5%_	0.326	0.040	-	-	0.374	0.017	-	-	0.345	0.029
PTV_48_ D_2%_	0.363	0.021	-	-	0.394	0.012	-	-	0.338	0.033
PTV_48_ D_1%_	0.393	0.012	-	-	0.415	0.008	-	-	0.362	0.022
PTV_48_ maximum dose	0.394	0.012	0.399	0.011	0.455	0.003	-	-	0.376	0.017
Spinal cord maximum dose	-	-	-	-	-0.347	0.028	-	-	-	-
Left lens maximum dose	-0.329	0.038	-		-0.368	0.020	-	-	-0.341	0.031
Local gamma passing rates										
PTV_67.5_ D_5%_	-	-	-	-	-	-	-0.314	0.048	-	-
PTV_67.5_ D_98%_	-	-	-	-	-	-	-	-	-0.346	0.029
PTV_54_ D_95%_	-	-	-0.363	0.021	-0.438	0.005	-0.366	0.020	-0.463	0.003
PTV_54_ D_50%_	-0.385	0.014	-0.321	0.044	-	-	-	-	-	-
PTV_54_ maximum dose	-0.359	0.023	-0.348	0.028	-0.337	0.034	-	-	-	-
PTV_48_ D_98%_	-	-	-	-	-0.347	0.028	-	-	-0.334	0.035
PTV_48_ minimum dose	-	-	-0.324	0.041	-0.354	0.025	-	-	-0.326	0.040
Body V_50%_	-	-	-0.362	0.022	-0.374	0.017	-	-	-	-
Spinal cord maximum dose	-	-	-	-	-0.320	0.044	-0.328	0.039	-	-

VMAT: Volumetric modulated arc therapy, DV parameter: Dose-volumetric parameter, *r*_*s*_: Spearman’s rank correlation coefficient, PTV_n_: Planning target volume with a prescription dose of *n* Gy, D_***n*%**_: Dose received by at least *n*% volume of the structure, V_n%_: Volume receiving at least *n*% of the maximum prescription dose.

### Correlations between the GPRs and the DV errors of the TrueBeam STx system

**Fig 1:**
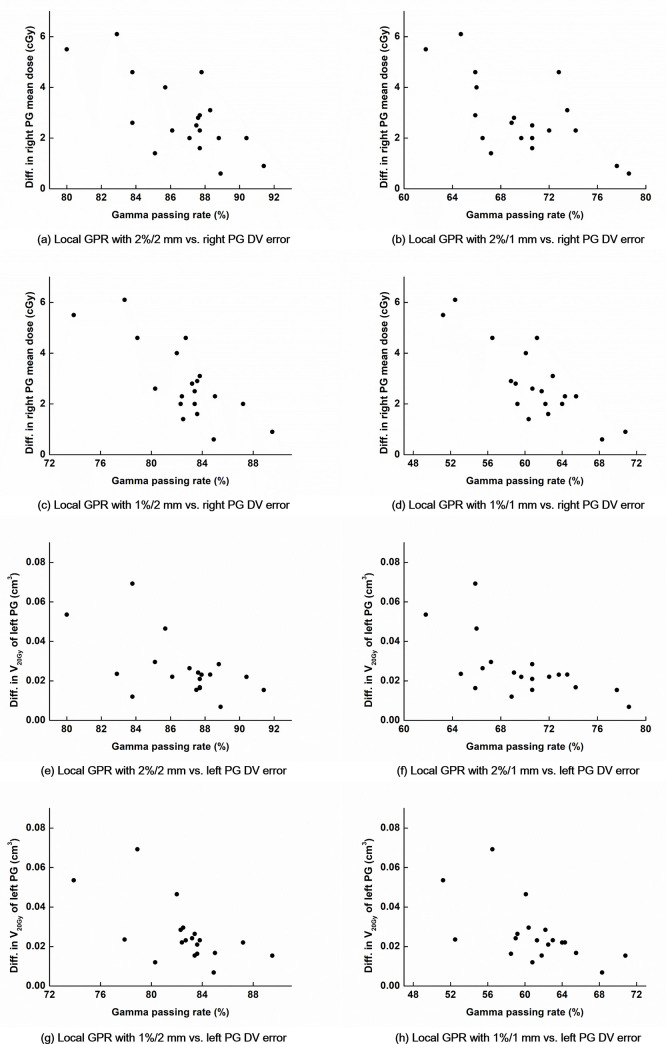
Differences in the dose-volumetric parameters of the parotid glands between the original volumetric modulated arc therapy (VMAT) plans and the plans reconstructed with the log files recorded during VMAT delivery according to the local gamma passing rates (GPRs) with various gamma criteria of the TrueBeam STx system. Differences in the dose-volumetric parameters between the original VMAT plans and the plans reconstructed with the log files recorded during VMAT delivery (DV errors) of the parotid glands (PGs) are plotted according to the local GPRs with various gamma criteria for the TrueBeam STx system. The DV errors of the mean dose of the right PG vs. local GPRs with the gamma criteria of 2%/2 mm (a), 2%/1 mm (b), 1%/2 mm (c), and 1%/1 mm (d) are shown. The DV errors of the V_20Gy_ of the left PG vs. local GPRs with the gamma criteria of 2%/2 mm (e), 2%/1 mm (f), 1%/2 mm (g), and 1%/1 mm (h) are also shown.

The average values of each DV error with the TrheBeam STx system are shown in S1 Table. Only the statistically significant correlation coefficients between the GPRs and the DV errors with the TrueBeam STx system are shown in Table 4 with the corresponding *p* values. Every correlation coefficients between the GPRs and the DV errors are shown in S2 Table. Among a total of 46 dose-volumetric parameters tested in this study, both the global and local GPRs with 1%/2 mm and the local GPRs with 1%/1 mm showed statistically significant correlations with DV errors most frequently (a total of 19 *r*_*s*_ values with *p* values less than 0.05). The highest correlation was observed between the global GPR with 2%/1 mm and the DV error in the V_20Gy_ of the left PG (*r*_*s*_ = -0.817 with *p* < 0.001). Compared with the *r*_*s*_ values of the Trilogy system, statistically significant *r*_*s*_ values were more frequently observed with the TrueBeam STx system and the absolute values of *r*_*s*_ of the TrueBeam STx system were higher than those of the Trilogy system. In most cases with the TrueBeam STx system showing *r*_*s*_ with *p* < 0.05, moderate correlations were observed (absolute values of *r*_*s*_ ≥ 0.4). The DV errors in the V_20Gy_ of the left PG showed strong or very strong correlations with the global GPRs with various gamma criteria (always showing absolute values of *r*_*s*_ > 0.66 except the GPRs with 3%/3 mm). The DV errors in the V_20Gy_ of both the left and right PGs and the mean dose of the right PG showed higher *r*_*s*_ values than the others with the local GPRs with various gamma criteria. Especially, the mean dose of the right PG showed strong correlations with every local GPR tested in this study except the GPR with 3%/3 mm (absolute values of *r*_*s*_ > 0.6). The DV errors in the mean dose of the right PG as well as those in the V_20Gy_ of the left PG with the TrueBeam STx system are plotted according to the local GPRs with various gamma criteria in Fig 1. As the GPRs increased, the DV errors decreased.

**Table 4:** Correlations of the gamma passing rates with the changes in the dose-volumetric parameters between the original VMAT plans and the plans reconstructed with the log files acquired during the VMAT deliveries of the TrueBeam STx.

DV parameter	3%/3 mm		2%/2 mm		2%/1 mm		1%/2 mm		1%/1 mm	
	*r*_*s*_	*p*	*r*_*s*_	*p*	*r*_*s*_	*p*	*r*_*s*_	*p*	*r*_*s*_	*p*
Global gamma passing rates										
PTV_67.5_ D_99%_	-	-	-	-	-	-	-0.427	0.050	-	-
PTV_67.5_ D_98%_	-	-	-0.487	0.029	-0.385	0.050	-0.516	0.020	-0.387	0.050
PTV_67.5_ D_95%_	-	-	-0.606	0.005	-0.517	0.021	-0.576	0.008	-0.497	0.026
PTV_67.5_ D_50%_	-	-	-0.543	0.013	-0.523	0.019	-0.479	0.033	-0.464	0.039
PTV_67.5_ D_5%_	-	-	-0.464	0.039	-0.499	0.027	-	-	-	-
PTV_67.5_ mean dose	-	-	-0.549	0.012	-0.540	0.014	-0.469	0.037	-0.452	0.046
PTV_54_ D_95%_	-	-	-	-	-0.382	0.050	-0.394	0.050	-0.435	0.050
PTV_54_ D_50%_	-0.577	0.008	-0.599	0.005	-0.612	0.005	-0.456	0.043	-0.502	0.024
PTV_54_ D_5%_	-	-	-0.490	0.028	-0.532	0.017	-0.405	0.050	-0.487	0.030
PTV_54_ D_2%_	-	-	-0.476	0.034	-0.424	0.050	-0.430	0.050	-0.409	0.050
PTV_54_ mean dose	-	-	-0.533	0.016	-0.556	0.011	-0.420	0.050	-0.460	0.041
PTV_48_ maximum dose	-	-	-0.549	0.012	-0.493	0.027	-0.497	0.026	-0.406	0.050
Body V_100%_	-	-	-0.516	0.020	-0.546	0.014	-0.464	0.039	-0.535	0.015
Body V_50%_	-	-	-0.497	0.026	-0.526	0.019	-0.488	0.029	-0.528	0.017
PG (L) V_20Gy_	-0.444	0.050	-0.699	0.001	-0.817	<0.001	-0.668	0.001	-0.785	<0.001
PG (R) V_20Gy_	-	-	-0.547	0.013	-0.439	0.050	-0.605	0.005	-0.568	0.009
PG (L) V_50%_	-	-	-0.558	0.010	-0.474	0.036	-0.530	0.016	-0.509	0.022
PG (R) V_50%_	-	-	-0.570	0.009	-0.513	0.022	-0.585	0.007	-0.618	0.004
PG (L) mean dose	-	-	-	-	-	-	-0.477	0.033	-0.427	0.050
PG (R) mean dose	-	-	-0.492	0.028	-	-	-0.566	0.009	-0.562	0.010
Local gamma passing rates										
PTV_67.5_ D_99%_	-	-	-	-	-0.419	0.050	-0.540	0.014	-0.454	0.045
PTV_67.5_ D_98%_	-	-	-0.453	0.045	-0.445	0.049	-0.632	0.003	-0.529	0.018
PTV_67.5_ D_95%_	-	-	-0.447	0.048	-0.463	0.040	-0.652	0.002	-0.586	0.008
PTV_67.5_ D_50%_	-	-	-	-	-0.401	0.050	-0.515	0.020	-0.441	0.050
PTV_67.5_ mean dose	-	-	-	-	-	-	-0.504	0.023	-0.424	0.050
PTV_54_ D_99%_	-	-	-	-	-0.408	0.050	-	-	-0.532	0.017
PTV_54_ D_98%_	-	-	-	-	-0.463	0.040	-0.488	0.029	-0.571	0.010
PTV_54_ D_95%_	-	-	-	-	-0.490	0.028	-0.408	0.050	-0.600	0.006
PTV_54_ D_5%_	-	-	-	-	-0.405	0.050	-0.578	0.008	-0.561	0.011
PTV_54_ D_2%_	-	-	-	-	-0.407	0.050	-0.664	0.001	-0.544	0.014
PTV_54_ mean dose	-	-	-	-	-	-	-	-	-0.502	0.024
PTV_48_ maximum dose	-	-	-0.463	0.040	-	-	-0.561	0.010	-	-
Body V_100%_	-	-	-	-	-0.551	0.012	-0.596	0.006	-0.638	0.003
Body V_50%_	-	-	-0.442	0.050	-0.478	0.033	-0.516	0.020	-0.577	0.009
Body mean dose	-	-	-	-	-	-	-0.498	0.025	-0.407	0.050
PG (L) V_20Gy_	-	-	-0.528	0.017	-0.642	0.002	-0.650	0.002	-0.674	0.002
PG (R) V_20Gy_	-0.510	0.022	-0.640	0.002	-0.525	0.017	-0.693	0.001	-0.591	0.007
PG (L) V_50%_	-	-	-0.460	0.042	-	-	-0.513	0.021	-	-
PG (R) V_50%_	-	-	-0.537	0.015	-0.475	0.034	-0.619	0.004	-0.580	0.008
PG (L) mean dose	-	-	-0.431	0.050	-	-	-0.577	0.008	-	-
PG (R) mean dose	-0.598	0.005	-0.603	0.005	-0.613	0.004	-0.657	0.002	-0.737	<0.001
ON (L) maximum dose	-0.458	0.042	-0.401	0.050	-0.449	0.047	-	-	-0.488	0.029

VMAT: Volumetric modulated arc therapy, DV parameter: Dose-volumetric parameter, *r*_*s*_: Spearman’s rank correlation coefficient, PTV_n_: Planning target volume with a prescription dose of *n* Gy, D_***n*%**_: Dose received by at least *n*% volume of the structure, V_n%_: Volume receiving at least *n*% of the maximum prescription dose, V_nGy_: Volume receiving at least *n* Gy, PG: Parotid gland, ON: Optic nerve.

## Discussion

In the present study, we observed moderate or strong correlations of the GPRs with some of the DV errors of the VMAT plans for H&N cancer with the TrueBeam STx system. Especially, the DV errors at the PGs showed higher correlations with the GPRs with various gamma criteria than those at the other structures. In contrast, weak or no correlations were generally observed between the GPRs and the DV errors with the Trilogy system, which is a C-series linac. The results obtained with the Trilogy system are consistent with those of the previous studies [4,5].

According to the values of the MCS_v_ and LTMCS, the modulation degrees of the VMAT plans with the TrueBeam STx system were almost the same as those with the Trilogy system and no statistically significant differences between them were observed (both with *p* > 0.05). In addition, the values of the local GPR_cal_ with 1%/2 mm and 1%/1 mm (both with *p* < 0.05) of the TrueBeam STx system were lower than those of the Trilogy system, which is reasonable because the mechanical errors (MLC positioning errors, gantry angle errors, and MU delivery errors) during delivery with the TrueBeam STx were much smaller than those with the Trilogy system. Nonetheless, both the global and local GPRs with various gamma criteria of the TrueBeam STx system were higher than those of the Trilogy system with statistical significance, except for the GPRs with 3%/3 mm (all with *p* < 0.05). This means the mechanical errors recorded in the Trilogy system during delivery were larger than those in the TrueBeam STx system, however, the actual dose delivery errors of the Trilogy system were larger than those of the TrueBeam STx according to the GPRs based on the measurements. This could be attributed to the more accurate VMAT delivery records in the log files of the TrueBeam STx system than those of the Trilogy system [19]. Previous studies showed that the most dominant mechanical error significantly affecting VMAT delivery accuracy among the three types of mechanical errors, *i*.*e*., MLC positioning error, gantry angle error, and MU delivery error, was the MLC positioning error [20–22]. The log file of the actual MLC positions during the VMAT delivery of the TrueBeam STx system is the Trajectory file, which is a record of the direct MLC position values with an update rate of 20 ms [19]. However, the actual MLC position log file of the Trilogy system is the DynaLog file, which is a record of the actual motor values with an update rate of 50 ms [10]. The actual motor values are converted to MLC positioning values using a conversion table (mlctable.txt) [10]. Therefore, the Trajectory file contains more accurate MLC positioning information than that of the DyanLog file owing to the small update rate and direct-record method. This could result in the accurate calculation of the DV errors with the TrueBeam STx system; therefore, higher correlations of the DV errors with the GPRs could be obtained with the TrueBeam STx system than with the Trilogy system.

The different results of the TrueBeam STx system from those of the Trilogy system in the present study might be attributed to the different types of the MLC systems (HD 120 MLC and Millennium 120 MLC) since the MLC leaf width and the design of the HD 120 MLC are different from those of the Millennium 120 MLC. This was not investigated in this study, which is a limitation of the present study, therefore, we will investigate this in the future by utilising VitalBeam (Varian Medical Systems, Palo Alto, CA, USA).

Despite the high modulation degrees (average MCS_v_ value less than 0.0014 and average LTMCS value less than 0.0006) of the H&N VMAT plans in the present study, all the VMAT plans utilised in this study were clinically acceptable, always showing global GPRs with 2%/2 mm higher than 90% [13,17,18]. Consequently, the magnitudes of the mechanical discrepancies between the original VMAT plans and the actual deliveries of the VMAT plans were small, which resulted in the small DV errors. Especially, the more accurate VMAT deliveries with the TrueBeam STx could be consistently identified from the higher GPRs, smaller mechanical errors recorded in the log files, and smaller DV errors than those with the Trilogy system. This was attributed to the more accurate VMAT delivery of the TrueBeam STx system than that of the Trilogy system, owing to the integrated mechanical parameter control system [19]. Despite the smaller ranges of the delivery errors with the TrueBeam STx system than those with the Trilogy system, higher correlations between the GPRs and the DV errors were observed with the TrueBeam STx system than with the Trilogy system owing to the reasons described above.

Previous studies that examined the correlations between the GPRs and the DV errors with IMRT plans concluded that the GPRs did not predict DV errors of clinically relevant DV endpoints [4,5]. In contrast, in the present study with VMAT plans using the TrueBeam STx, the GPRs with 1%/2 mm and 1%/1 mm showed moderate or strong correlations with some of the DV errors, which is the first report to the best of our knowledge. The GPRs showed strong correlations with some (but not all) of the DV errors. Since the gamma index method quantitatively evaluates the accuracy of VMAT delivery with a single value, *i*.*e*., the GPR, it can only be recognised whether the overall VMAT delivery accuracy would be high or not with the gamma index method. In other words, if the GPR is low, we would be aware that the VMAT delivery to a patient would be inaccurate but we would not know the location of the inaccuracy in the patient’s body, *i*.*e*., spatial information of the errors in the patient’s body would not be provided by the GPRs. This is a limitation of the plan verification with the GPR. Therefore, as the AAPM TG-218 protocol recommended, in addition to the GPR, various types of information provided by the gamma index method such as gamma value and gamma map should be examined comprehensively [23]. According to the results of the present study, the GPRs of the VMAT plans with the TrueBeam STx system could indicate the occurrence of DV errors during VMAT deliveries; however, they could not indicate where and what kind of DV errors would occur in a patient’s body.

In the present study, the local GPRs with 1%/2 mm and 1%/1 mm showed strong correlations with the DV errors at the PGs. This appears reasonable as the DV constraints of the PGs are generally difficult to satisfy because the PGs are generally overlapped with or extremely close to the PTVs (PTV_54_ or PTV_67.5_). Therefore, steep dose gradients were generally generated between the PGs and the target volumes by highly modulated photon beams, which is highly sensitive to the uncertainty of VMAT delivery [23–25]. If there is a discrepancy between the original VMAT plan and the actual delivery of VMAT at the steep dose gradient generated in or near the PGs, DV errors would occur at the PGs [23–25]. In this respect, strong correlations were observed between the GPRs and the DV errors of the PGs in the present study.

The limitation of the present study is that we could not include the clinically unacceptable VMAT plans, which failed in the gamma evaluation owing to the extremely high modulations. Therefore, we could not determine the tolerance levels of the gamma index method based on the DV errors of clinically relevant DV endpoints in this study. Another limitation of the present study is that the number of VMAT plans analysed in this study was limited, *i*.*e*., only 20. Besides the number of VMAT plans, the tumour site of the VMAT plans in the present study was limited by the analysis of only H&N VMAT plans. We will conduct further studies in the future to overcome the limitations of the present study.

In the present study, we demonstrated that the GPRs with tight gamma criteria could predict some DV errors by utilising a linac system whose log record system during VMAT delivery is accurate. Therefore, the gamma index method is worthy to be performed before the deliveries of VMAT plans to patients for accurate patient treatment. In addition, the gamma index method is an independent verification method for the accuracy of VMAT plan delivery based on measurement. In the present study, although the GPRs with 1%/2 mm and 1%/1 mm could predict some DV errors of clinically relevant DV endpoints of VMAT, they do not provide spatial information of the DV errors. Therefore, when utilising the gamma index method, comprehensive analysis through GPR evaluation including gamma value and gamma map analyses should be performed.

## Supporting information

S1 Table Average values and corresponding standard deviations of each dose-volumetric parameter error in the present study. (XLSX)

S2 Table Every Spearman’s correlation coefficient examined in the present study between the dose-volumetric parameter errors and gamma passing rates. (XLSX)
